# Administering Antineoplastic Parenteral Cancer Therapy in the Patient's Home: Perspectives of Oncology Nurses—A Descriptive Qualitative Study

**DOI:** 10.1002/nop2.70891

**Published:** 2026-10-06

**Authors:** Tranberg Albin, Kälvemark‐Sporrong Sofia, Paulsson Mattias

**Affiliations:** ^1^ Department of Women's and Children's Health Uppsala University, Akademiska Sjukhuset Uppsala Sweden; ^2^ Department of Pharmacy, Uppsala Biomedical Center Uppsala University Uppsala Sweden

## Abstract

**Aim:**

To explore oncology nurses' perspectives on performing cancer treatments with parenteral antineoplastic agents in patients' homes.

**Design:**

A descriptive qualitative study utilizing interviews.

**Methods:**

Semi‐structured interviews were performed with oncology nurses at a hospital oncology daycare ward between October and November 2022. The interview data were inductively analysed with thematic content analysis.

**Results:**

Five themes were identified: (1) Establishing rapport in the home environment, (2) The complexity of a home, (3) Adverse events—not practicalities, (4) Solitary nursing and (5) Planning enables performance. The nurses believed patients would benefit from home treatment with antineoplastic agents but were concerned about adverse events and working alone. For successful home treatments, nurses believed sufficient resources, education and support were necessary. Practicalities such as transporting, administering and waste handling were not considered complex. Neither did the antineoplastic agents handling seem problematic.

**Conclusions:**

Although positive, especially from the patient perspective, the nurses identified concerns that need to be addressed before implementing oncology hospital‐at‐home services.

**Implications:**

Working in different contexts than the hospital can lead to extended roles and further responsibilities for oncology nurses and possibly facilitate more person‐centred care.

**Impact:**

This study explored oncology nurses' perspectives on performing parenteral cancer treatments with antineoplastic agents in patients' homes. In general, the nurses were positive, but challenges were identified. The study provides insights into how healthcare developers can incorporate the needs of nurses when designing future hospital‐at‐home programmes.

**Reporting Method:**

COREQ guidelines were used.

**Patient or Public Contribution:**

No patient or public contribution.

**What Does This Paper Contribute to the Wider Global Clinical Community?:**

This paper can be used to create tools addressing barriers nurses might have working with hazardous antineoplastic agents in hospital‐at‐home environments. It emphasizes the importance of education, resources and support to provide nurses with an adequate and safe working environment, improving patient experiences and safety.

## 1 Introduction

The survival rate of cancer increases with the development of efficient treatments resulting in an increased oncology workload for healthcare professionals (HCPs) (Garattini et al. 2022). Nurses comprise a major part of the healthcare system and by giving this profession more assignments outside existing healthcare facilities, more patients can likely be treated and cared for.

Current research lacks information on nurses' experience from and attitudes towards providing hospital‐at‐home (HaH) care with antineoplastic agents. To address this knowledge gap, the purpose of this study was to assess Swedish oncology nurses' perspectives on performing cancer treatments with antineoplastic agents in patients' homes.

## 2 Background

With advanced diagnostic techniques and modern treatments, the life expectancy of cancer patients increases and often, the patients can maintain a normal lifestyle for several years. It is essential that these patients receive good support, treatment and guidance from HCPs and that healthcare systems actively work with evolving cancer care.

The standard methods for treating cancer include radiation, surgery and drug therapy, with frequent instances of combination (Stevens 2023). However, many antineoplastic agents (ANPA) are based on hazardous drugs (HD) and require careful handling because their attributes meet at least one of the following criteria: carcinogenicity, teratogenicity, reproductive toxicity, organ toxicity at low doses or genotoxicity (Connor et al. 2016).

Several studies have demonstrated contamination of healthcare facilities with ANPA (International Society of Oncology Pharmacy Practitioners [ISOPP] 2022; Roussel et al. 2019). Exposure to ANPA has been connected to several short‐ and long‐term negative consequences (Mahmoodi et al. 2017; Moretti et al. 2015) and exposure to cytotoxic agents is connected to an increment in risk for spontaneous abortions in females (Liu et al. 2023). In addition, ANPA exposure is also associated with a greater extent of genotoxicity (Villarini et al. 2016).

To help protect HCPs several guidelines are developed (Friese et al. 2020; ISOPP 2022; Connor et al. 2016; Power and Coyne 2018). However, a systematic review showed that guidelines mostly covered the handling of HD drugs in health centers, only 19.5% include the home setting. In addition, there was only one international consensus guideline covering the entire process of HD drug manipulation (Bernabeu‐Martínez et al. 2018).

The definition of HaH is not fully established, but it can be described as a healthcare model where hospital‐level healthcare is performed in the patient's home (Leong et al. 2021). Generally, there are two different goals: early hospital discharge with support or avoiding hospital admission (Leong et al. 2021). A meta‐analysis concluded that HaH can reduce mortality in all age groups, and that it can reduce costs and readmission rates (Caplan et al. 2012). A systematic review of reviews concluded that HaH, especially admission avoidance, can improve clinical outcomes such as readmissions and mortality (Leong et al. 2021).

Although previous studies have suggested HaH as a potential alternative to regular (hospital) care, parenteral ANPA administered in a HaH setting is not yet fully established, and there is a lack of guidelines, standard procedures and definitions (Evans et al. 2016). In addition, the evidence regarding parenteral ANPA in HaH is disputed because of the heterogeneity in studies regarding outcome measurements, study design, interventions, cost measurements and prevalence of bias (Corbett et al. 2015; Evans et al. 2016; Mittaine‐Marzac et al. 2021). For example, a review from 2015 states that no improvements in quality of life (QoL) or psychological and‐ clinical‐outcomes could be seen, however ANPA in HaH makes ‘maintaining normality’ in life with cancer easier (Corbett et al. 2015).

Most programmes that offer parenteral ANPA in HaH are structured similarly; the first administration of treatments takes place at the clinic, and the successive treatments are provided by oncology or ‘advanced homecare’ nurses in the patient's home (Evans et al. 2016). Hospital pharmacies usually compound or prepare the ANPA, pack them and ship them to the patient by couriers or with the nurses. However, relatively little is known about safety and training protocols for nurses to follow when performing ANPA in HaH.

In Sweden, healthcare is managed independently among 21 local regions and 290 municipalities. Although they are independent, they adhere to the laws and regulations instituted by the Swedish parliament. The prevalence of cancer in 2022 was 312,271 in women and 290,703 in men (Socialstyrelsen (The National Board of Health and Welfare, Sweden) 2022) and between 1980 and 2016 the ten‐year survival rate improved from under 50% to over 60% for both genders (Regionalt cancercentrum Mellansverige (Regional Cancer Centre in Sweden, Middle Sweden) 2021). In 2015, a national programme for centralized standardized patient cancer care pathways was established due to the long and varying waiting times for patients within the field (Wilkens et al. 2016). The programme aims to shorten the queues through standardized care pathways (SCP) tailored for different cancer types. Each SCP contains a set of indications, diagnostic procedures and treatment options. The treatment options vary between each SCP but usually contain a mix of chemotherapy, radiotherapy and surgical procedures. All SCPs have allocated timeframes the cancer caregivers are recommended to adhere to. Nevertheless, only 51% of cancer patients in 2020 went through the time frame specified for each SCP (Regionala cancercentrum i samverkan (Regional Cancer Centres in Sweden) 2021). Although there are many SCPs, none of them routinely involve a HaH approach where parenteral infusions or injections of ANPA are provided in the home by either relatives or nurses. In general, the ANPA treatments are given in oncology wards.

In the hospital of this study, there is interest in investigating the effectiveness of providing ambulatory policlinic cancer care closer to or within the patient's home. The rationale behind this was the 5% annual increase in cancer patients observed at the hospital, resulting in an increased burden for HCPs. As part of a development project exploring ambulatory oncology care models, patient interviews and surveys were conducted (Ödman et al. 2022). The results showed that about one‐third of cancer patients would prefer care in or closer to, the home instead of at the hospital. In addition, more than half of the patients visited the hospital over 30 times for treatment and medical assessments. Approximately 20% of the patients travelled 1–2 h to reach the hospital. In a survey with the nurses at the oncology daycare ward, the nurses replied that more uncomplicated treatments, such as immunotherapy, would work outside of the hospital but that moving all ‘simple’ treatments would only leave the ‘harsh’ treatments at the hospital, making the working environment harder and monotonous (Ödman et al. 2022). They also saw a need to develop routines and procedures to ensure safety when treating patients at home and that a standby oncology treatment team should be available if adverse events or accidents occurred.

## 3 Aim

Inspired by the increased interest in developing HaH oncology services, the aim of this study was to investigate oncology nurses' perspectives on performing cancer treatments with ANPA in the patient's home.

## 4 Methods

Since the project aimed to explore views and thoughts about a relatively unstudied phenomenon, a qualitative study design was chosen, using semi‐structured interviews. This was to understand several aspects of nurses' understanding and perspectives of the issue. To ensure sufficient reporting, the study was guided by the Consolidated Criteria for Reporting Qualitative Research checklist (COREQ) (Tong et al. 2007), the checklist is available as Data S1.

### 4.1 Authors' Preunderstandings

The first author is male, and at the time of data collection was a Master of Pharmacy Student. He had no previous experience conducting qualitative studies or interviews.

The second author is female, and a professor with a background in social science. She has experience of research on healthcare professionals, including nurses and their work environment. She has extensive experience of conducting qualitative research.

The last author is male, an associate professor and a practising clinical pharmacist, but not at the clinic investigated in this research. He has extensive knowledge on compounding, but not clinical oncology. He has some previous experience of qualitative research.

The authors' epistemological standpoint is that knowledge is subjective, affected and shaped by personal experience, views, expectations, values and the surrounding social environment. Hence, the data analysts' views will also be mirrored in the result, and no objective truth can be defined or derived from the material. Therefore, the choice of method and the result from data analysis depend on the theoretical framework and the preunderstanding of the one performing it; in other words, the researcher will be an active creator of the result. Further reflections on how our pre‐understandings shaped the data collection and analysis processes are summarized in the discussion section.

### 4.2 Study Setting and Recruitment

The study population consisted of nurses working at a policlinical daycare oncology ward at a large hospital in Sweden. The nurses were knowledgeable in ANPA and the prescribing software used within oncology care. Therefore, they can be viewed as candidates to perform ANPA in HaH in the future. Inclusion criteria for this study were nurses who handled ANPA and worked clinically. Their responsibilities include scheduling and coordinating appointments with oncologists, treatment, radiology, overseeing lab results, answering questions related to treatments and drugs, medical assessments, follow‐ups and checking for adverse events. In addition, they handle and inform patients about the ANPA used, as well as order ANPA that require compounding from the hospital pharmacy.

To assist with recruitment, the head nurse of the ward informed the nursing staff about the study during daily meetings. The first author also informed the staff about the study while shadowing the nurses to gain familiarity with their daily clinical routines (see Section 4.3.1).

After the nurses had been informed about the study, the head nurse used the ward staffing schedule to identify nurses with one‐hour time slots characterized by low clinical demands. This was done to ensure that interviews could be conducted during office hours without compromising the quality of patient care; the head nurse invited available staff using this convenience sampling approach. However, within these available slots, nurses with different personal backgrounds (age, gender), education levels and work experience were invited to facilitate variation (Brinkmann and Kvale 2015). Before every interview, the interviewee received written and oral information about the study from the interviewer (first author), face‐to‐face. The first author emphasized that participation was entirely voluntary and that they could decline to participate or withdraw from the study at any time if they wished. Additionally, they were informed that they could reschedule the interview outside office hours if they wanted to.

The first author had previously met some of the interviewees when working at another department/clinic in the hospital. The interviewer presented himself as a student and explained that he was not directly associated with the hospital's homecare project. Furthermore, he clarified that neither the head nurse nor the directors of the hospital homecare project would have access to the interview transcripts.

### 4.3 Data Collection

#### 4.3.1 Interview Guide Development

The interview guide was developed from existing literature related to oncology HaH programs (Burns et al. 2020; Corbett et al. 2015; Corrie et al. 2013; Evans et al. 2016; Mittaine‐Marzac et al. 2021), internal hospital routines and guidelines. Inspired by Kallio et al. (2016) the interview guide was restructured and discussed between the authors to adjust for potential leading or incomprehensible questions. The first author shadowed nurses 1 day at the department to socialize and potentially gain trust. In addition, notes were taken regarding the vocabulary used in order to use the same terminology during the interviews, thereby facilitating participant‐oriented interviews (DeJonckheere and Vaughn 2019). Thereafter the revised interview guide was sent to two policymakers with central positions at the hospital to receive assessment from people with operational knowledge within the oncology field. No additional changes were made to the interview guide.

The final interview guide consisted of 10 sections. The first contained demographic questions (work experience, education). Since none of the participating nurses had yet performed ANPA in HaH, the second part was constructed to stimulate reflection on and descriptions of how drugs are currently handled at the department. This was done to establish trust, make the interviewee comfortable and stimulate the interviewee to reflect upon their drug handling (Brinkmann and Kvale 2015; DeJonckheere and Vaughn 2019; Kallio et al. 2016). Thereafter, the core of the interview questions followed and at the end, the interviewer summarized the topics covered and invited the interviewee to give additional information. See Table 1 for all sections of the interview guide. The order of topics was adapted depending on the interviewees' answers and interviewees were encouraged to visualize themselves performing practical tasks in the home such as preparing calcium‐folinate injections and checking the backflow of ‘PICC lines’. This was done so that nurses could transfer their current experience into hypothesized situations doing similar tasks but in the patient's home. Follow‐up questions were used extensively.

**TABLE 1:** The ten sections of the interview guide.

Major part	Example questions
Initial chat & collection of demographic information:	How many years have you worked in the field of cancer? Have you previously worked with home healthcare/HaH?
Warm‐up	Can you tell me what a typical workday looks like? If we now focus on drugs, how do you handle them on a typical workday?
Bridging questions to move focus to HaH and cancer drugs	If you imagine doing the same work with medication/drugs that you do today, but in patient's home. What do you think about that?
Transport of medication to the patient's home	If intravenous cancer drugs were to be used in a home, transport of the drugs may be required from the hospital to the patient's home. What do you think about transporting cancer medication to and from the patient? How would you feel about transporting cancer drugs to and from the patient?
Preparation of cancer drugs in the patient's home	Some medications are not adapted for transport and may need to be mixed and prepared in the patient's home. What do you think about mixing and preparing medication in the patient's home? How would you feel about mixing and preparing medication at a patient's home?
Informing & educating about cancer medication in the home	What do you think about providing patients with information, instructions and education about medication and treatment in their home? How do you feel about answering patients' questions in their home?
Administration of cancer drugs in the home	What do you think about administering cancer medication in the patient's home? How do you feel about administering cancer medication in the patient's home?
Handling of cancer drug waste in the home	What do you think about handling medication waste in the patient's home? How do you feel about handling medication waste in the patient's home?
Conclusion and discussion	What do you think are the biggest advantages for you as a nurse when performing cancer care in a patient's home? What are the biggest barriers for you as a nurse to perform cancer care in the patient's home? How do you see cancer care in the home as part of your professional role? What responsibility do you feel your professional role has?
Disengagements and end of interview	Closing: Now my interview is coming to an end, but is there anything you would like to add regarding the handling of cancer drugs in patients' homes? Summarizing of conversation Closing and turning off the microphone Post‐interview discussion and follow‐up on how they thought the interview went and felt.

After the second interview, changes were made to the written information about the study. The word ‘concerns’ was removed from the part informing about the study to not overly lead interviewees to only focus on concerns.

#### 4.3.2 Data Collection

The first author conducted all the interviews face‐to‐face between October and November 2022. The interviews took place in available, empty offices at the ward; no one except the first author and the interviewee was present during the interviews. No repeated interviews were performed.

The interviews were audio recorded and lasted 25–56 min, with an average of 38 min. After each interview, the interviewer's reflections on the interview were documented to improve the quality of the following interviews, promote reflection and bring context to the later analysis of transcripts (Brinkmann and Kvale 2015; DeJonckheere and Vaughn 2019). All interviews were transcribed verbatim by the first author within 10 days of each individual interview; however, the formal analysis was not performed until all interviews had been transcribed.

To enhance credibility, member checking on the transcripts were used, and all interviewees were asked if they could read their transcripts after the interviews. This was done to check for potential errors, add or recall information and confirm that the information in the transcripts was seen as true and acceptable. Five out of nine interviewees agreed to do this and all agreed to the content of the transcripts (Amin et al. 2020).

### 4.4 Data Analysis

The data analysis method was thematic analysis (TA) (Braun and Clarke 2022). This was used in an inductive way. The coding was performed in Microsoft Word and Excel. Member checking the findings from the data analysis was not performed. The first author conducted the majority of the analysis.

In step one, all the transcripts and their respective ‘post‐interview notes’ were read multiple times to gain a deep familiarization and understanding of the transcripts. In step two, each interview transcript was searched for segments considered relevant to the aim and research questions of the study, and each segment was assigned a code. In step three, codes that seemed to have the same underlying meaning were merged and then sorted into primary themes. In step four, all themes were reviewed to evaluate if they properly reflected the underlying codes and data segments; this was repeated until the first author concluded that they captured the variety of the data. The transcripts were then re‐read multiple times to ensure that themes accurately captured the meaning of transcripts and to identify data that the themes failed to capture. In step five, themes were named and compared to make sure no unclear overlap between them existed. In step six, a summative description and narrative story of each theme was written. Illustrating quotes were added from the transcripts to provide evidence and context to the themes. Since the data analysis was conducted in Swedish, the quotes presented in the report were translated into English after the analysis. The validity of the translations was reviewed and approved by all authors.

To strengthen the data analysis and increase confirmability, credibility and reduce bias in the data analysis, a coding workshop and an audit were conducted. The coding workshop included the first and the last author and a Master of Pharmacy student between steps two and three. In the workshop, the two most different transcripts were discussed after individual coding by all three participants. The codes originating from the three coders were compared, and discrepancies were resolved by discussion to reach a consensus. Ultimately, the coding workshop did not result in any discoveries of new codes or perspectives; this might be because all the coders had similar backgrounds.

The audit took place in the transition between step three and four, with the second author. The second author coded by hand the two most different transcripts and the connected extracts from the first author's research diary. Thereafter, a consensus meeting was held, including discussions of similarities and differences between the two codings and how themes could be formed and narrated. Hence, the audit added variance to the data analysis. The consensus meeting resulted in minor changes to the original themes and the addition of a new theme.

All cases, also differing or opposing, were included in the analysis and are part of the results. No participant validation of the result was conducted. To ensure transparency, tables of themes, codes and original Swedish quotes with English translations are available as Tables S1–S5.

### 4.5 Information Power

Regarding sample size, this was based on Malterud et al.'s reasoning about information power (Malterud et al. 2016). The aim of the study was relatively narrow, and the respondents were dense; that is, they had experiences related to the subject, both allowing the sample size to be smaller. The interviewer was novel to interviewing, possibly limiting data richness; however, the interview content was rich, suggesting a strong dialogue (Malterud et al. 2016). Finally, the data analysis was inductive and exploratory, suggesting a need for more interviews to capture the variety of existing perspectives. However, a relatively high proportion of nurses from the ward were interviewed, and the nurses included had a diverse range of experience.

## 5 Findings

### 5.1 Study Population and Demographics

A total of nine nurses were asked to participate in the interviews and all agreed to do so. The analysis was based on all participating nurses. See Table 2 for demographics of the interviewed nurses.

**TABLE 2:** Demographic characteristics of the interviewed nurses.

Demographics	Value (*n* = 9)
Men	3
Proportion of ward nurses interviewed	75%
Education	
Bachelor of Science in Nursing	9
Postgraduate Diploma in Specialist Nursing	0
Experience (years)	
Experience of working as a registered nurse (mean, min‐max)	16.2 (2,5–24) years
Experience of working with cancer therapy (mean, min‐max)	10.6 (1–23) years
Previous work with homecare	3

The analysis resulted in 5 themes: (1) Establishing rapport in the home environment, (2) The complexity of a home, (3) Adverse events – not practicalities, (4) Solitary nursing and (5) Planning enables performance.

### 5.2 Theme 1: Establishing Rapport in the Home Environment

The data indicated that HaH was perceived as something positive, facilitating a deeper understanding of the patient's needs. Most of the nurses portrayed the home environment as relaxing, promoting undivided dialogue between patient and nurse, and a way of integrating advice and information into the patient's own environment. In comparison, the nurses seemed to believe that the hospital environment limited patient contact, restricting the depth of it. In general, it appeared that educating patients about their cancer treatment in the home was regarded as a higher quality of caregiving, for the individual patient, compared to the hospital:

Nurse 1 In some way, it feels like…. I don't think you have the same level of stress as when you are working in a hospital setting. In a hospital, you have to divide your attention among many patients and tasks, whereas when you are working with a patient in their home, you can focus all your attention on just that one patient. This is beneficial for the patient, as they have your undivided attention and can feel like they are being fully cared for. It's a good feeling, knowing that you can just focus on that one patient and nothing else.

The nurses frequently expressed that HaH would enable a greater understanding of the patient's health, life situation and environment. Easing the discovery of need for additional nursing and ways of tailoring the care to the patients' specific needs. This increased personal understanding was portrayed as essential and an eclipse of providing good care, it seemed to be regarded as rewarding for the nurses:

Nurse 5 The patient would have other options for how they want to receive treatment when they get their medication, sitting, lying down, being in their bed, sitting in their armchair, watching TV, ehmmm… but giving the medication I don't think would be any different for me, whether at home or here [at the hospital], but in the home I would feel like ‘how nice, now the patient can sit and watch their TV show while they receive this treatment’

The nurses often believed that immobile or palliative patients would benefit most from receiving HaH, as it could allow more time spent with family due to reduced travel. However, even if most nurses believed that patients would benefit from HaH, some pointed out that the patients themselves should decide which care they prefer to receive.

### 5.3 Theme 2: The Complexity of a Home

Although the patient might benefit from HaH, the nurses thought HaH could be unhygienic compared to the hospital, especially when preparing drugs. This was clearly perceived as troublesome for retaining patient safety and a proper working environment. It was emphasized that clean surfaces were needed when preparing and handling drugs in a home. However, the nurses were willing to prepare drugs in the home if they received training and access to proper preparation equipment such as syringes, closed system transfer devices, sterile sodium or glucose solutions.

Nurse 5 And you still want a clean and good surface that is easy to disinfect and so on, so it would be a little more practical to have access to a little more space in the patient's home.

Some nurses believed that patients could view HaH as burdensome and compromise their privacy. The nurses often believed that giving the patient (and relatives) responsibility for ‘nursing tasks’ in their home, such as handling and storing equipment, drugs and waste, can sometimes be unwanted and burdensome for the patient.

Nurse 4 No, but maybe it's better bringing equipment so that the patients don't have the responsibility for it, because then it becomes difficult for the patient. But maybe as a nurse, I don't know? Have a medication box from the hospital pharmacy and drive to the place where it should be, so they have the infusion in place. Maybe you can't…, but no…. I don't want it to become a burden for patients and their loved ones.

In addition, proper patient—nurse communication was seen as needed to ensure a suitable working environment in the home without compromising the patient's privacy. Some worried that a HaH setting could cause patients to feel obliged to treat the nurses as guests, altering the nurse—patient relationship.

Nurse 6 One potential downside is that patients may feel like they have to offer something in return for my visit—like they are receiving a social visit at home, rather than receiving medical treatment. This can create a period of adjustment for both the patient and me, and there is a risk that our relationship might not be as good as it could be. Some patients might feel like they have to offer snacks or other refreshments when I visit, and this could create unnecessary stress for them. These are the kinds of things that I worry about when it comes to providing treatment in the home setting.

Regarding waste handling, many nurses replied that the responsibility should not be placed on the patient since it could lead to unwanted drug exposure of family members, pets and the home. Most nurses believed it would be best to immediately bring it back to the hospital in safe portable containers after finishing the treatment.

Nurse 2 We have very secure systems now with re‐sealable bags that we can use for cytostatics and such stuff, but we've actually done tests here at the hospital and seen that there are still traces of cytostatics, for example/…/ on these boxes and even to a certain extent on some of the carts. I'm thinking there could be several people living in the household and I think the best thing to do would be if we just brought it with us when we're done and take it back to the hospital.

Ergonomics was a frequent topic in the interviews. The nurses described that a home can be narrow and difficult to work in since patients might be unsuitably positioned. In contrast to the hospitals, where there is access to open spaces and aiding equipment. It appeared as some nurses found it complex, while others thought it was manageable with practise.

Nurse 4 What I believe is that ergonomically it will be harder, because here we can adjust the height of a hospital bed and we can have a stool or something. At home, it is likely that you will sit like a folding knife. Often, they may sit on the couch or in their own bed, which is not adjustable in height, or on a kitchen chair. it may be a challenge for many if they do it all the time… it can be tiring for the back, for example, or they may have to sit on their knees or something like that.

Many nurses believed that hospitals at home would require the nurse to carry around a lot of equipment, something that appeared to be slightly burdensome:

Nurse 1 It's with all the stuff with the drip stand, but all those things… those that you need to be able to administer. It's a hassle and we already see that with those who go to [neighbouring city hospital] who have to drag bags and everything around. And you also need an emergency bag and such things, so it's a lot but if you just look at the medication itself then it's not a big problem, but it's everything around that is needed.

### 5.4 Theme 3: Adverse Events—Not Practicalities

This was the most prominent theme; it captures the fear of adverse reactions when administering ANPA in the home. The nurses described practicalities of HaH, ‘set up an administration, preparing, transportation, etc.’ as something every nurse could perform. However, the difficulty they saw was handling the side effects and complications that ANPA can bring. Many of the less experienced nurses described that they lacked the education and experience to manage adverse events in a home, and it would be stressful to accurately handle adverse events without the help from colleagues. Especially since you would lack the resources associated with the hospital environment, including immediate assistance.

Nurse 3 Technically speaking, administering a drip at home shouldn't be a problem. It's not hard to put in a needle or use a PICC‐line or other equipment. It's pretty simple, really. The problem is the side effects and other issues that can come up with home infusions—things like allergic reactions… So, as far as I can see, the drip itself shouldn't be a problem to give at home.

The reactions were often portrayed as unpredictable even though ‘unharmful’ drugs such as monoclonal antibodies were used, and many nurses often described worst‐case scenarios where patients at the hospital had had adverse reactions even though they passed all the criteria for receiving treatment and had undergone the same treatment before.

Nurse 4 The difficult part is that things can happen to patients that can cause them to feel unwell even though they have received at least one treatment before… Some medications are known to cause reactions and some medications may work well for many people, but suddenly… then many times later, suddenly they react. And then you might not want it at home.

In addition to this, many nurses viewed it as a decision‐making problem, even though they might be experienced enough to revoke adverse reactions in the home. The question of who would be responsible for prescribing the drugs and procedures used was something concerning. Will there be a physician or only yourself? Who will be accountable if something goes wrong?

Nurse 1 Experience allows us to do a lot ourselves, but really you should have a doctor who prescribes everything as well. We know what to do and it feels safe if you are experienced and work. So yeah, if you don't have the experience, you don't have the knowledge and understand what can happen.

When talking about administering ANPA in the home, many nurses expressed that it would be suitable only to give low‐risk drugs, with few adverse events, to ensure patient safety and a proper working environment. However, they also believed that few drugs would satisfy this criterion. In addition, many believed that you needed to primarily give drugs with short infusion times to make HaH resource effective so you would be able to treat more than just a few patients per day.

Nurse 9 No, but I believe that there is one thing to consider. It is important to consider which medications carry a risk of reactions, and to try to remove as many of them as possible from home treatments. This is beneficial for both the patients and the healthcare professionals working with them. This is an important consideration.

### 5.5 Theme 4: Solitary Nursing

The nurses all expressed that the absence of direct access to colleagues would impact their work if working in the patient's home. Even though the nurses work relatively independently at the hospital, they described that communicating and collaborating with colleagues was a significant part of their work and important to ensure proper care and patient safety, especially for more inexperienced nurses. The availability of direct support was often considered a ‘safety net’ where colleagues could help with everything from patient assessments to providing acute help in case of adverse reactions.

The constant availability of an acute support team at the hospital was also seen as reassuring; knowing that you could receive immediate help in acute situations was described as relieving. The lack of this in a HaH environment appeared to be stressful and uncomfortable.

Nurse 2 What I think is added is more about…. if something more acute happens, that ehhhmmm if something acute happens here [at the hospital] then a couple of nurses rush in and the on‐call doctor can be there in 5 minutes, but that won't be the case if you're in someone's home.

Not just the lack of acute help from colleagues was portrayed as lacking in the home, but also the ‘normal’ communication and discussions with colleagues that occur naturally throughout the day. The opportunity to double‐check something and ask questions about treatments or side effects to a more experienced colleague was considered important. The colleagues at the ward were often viewed as a nearby source of collective knowledge that was necessary to be able to work with the multiple drugs and treatment regimens that exist in a polyclinical environment. In addition, there appeared to be an underlying uncertainty among the nurses concerning confidence in making decisions alone. Many nurses believed they needed more education and experience and that they depended on the collective knowledge that a group of colleagues can form together.

Nurse 7 Another aspect when working at someone's home is that I have no colleague to discuss things with. We discuss a lot of things here, I ask about something practically every day, ehmmm and so do those who have worked here for 20 years, asking about things. But the longer you have worked, the more side effects and reactions you have seen and can feel confident in answering the patient about. Because I do not always do so based on my own knowledge!

In terms of responsibility, many thought the responsibilities would not be changed in the home compared to the hospital. Still, the possibility of being responsible and ensuring the patients' safety and making the correct decisions and patient assessments would be more challenging while being alone without support from colleagues, especially in an acute situation. And who would be responsible for the patient's fate if something unpredictable happened?

Nurse 7 Yes, it is a very big responsibility to… that everything will be right of course. That I should be knowledgeable enough to take care of both the pre‐treatment or what to say, with assessment of symptoms, side effects, general condition at the moment, and then that I am confident in giving the treatment at the moment, and of course then giving the treatment correctly and disconnecting and giving all aftercare as well. So it is a very big responsibility… It is also the case here of course, but you stand more on your own at home… So it is!, ahh it is a big responsibility.

### 5.6 Theme 5: Planning Enables Performance

This theme captures the need for proper planning, resources, equipment, education and support when conducting HaH. When asked about potential practicalities (transporting drugs, preparing, administering and waste handling, etc.) that would be a part of ANPA in HaH, the nurses did describe concerns (themes 2, 3 and 4). Still, they made multiple suggestions for solutions and suggestions for what they thought would work best—everything from checklists, working in pairs, bringing emergency medical kits, using portable waste containers, portable tables and protective pads, having available spill kits, to having dedicated backup teams.

Nurse 7 No, but a foldable camping stool or something, something that you can sit fairly comfortably on. It will also be that… Here we can raise the bed, we can raise it if we don't get backflow, for example, in a vein port, if so we can tilt the hospital bed so that they lie upside down. It is designed to be user‐friendly in the hospital. Of course, that can't be the case in a home! We can't take hospital beds to the patient home! But then we'll have to make do with a good stool for ourselves, that's it.

Something many nurses mentioned was the usage of portable and adjustable chairs to help ensure proper working ergonomics. To ensure safety, mobile ‘acute care bags’ that could be used in case of drug reactions were suggested. For transport, robust sealed bags were proposed, like those already used for transporting compounded drugs to other external hospitals. Regarding preparation in the home, the nurses believed that closed‐system transfer devices would protect against exposure and contamination, but mainly emphasized that the standard procedures for preparing in the home needed to be safe. If preparation of more complex drugs, compared to the ones they prepare today, would be required in a HaH setting, they believed sufficient training was needed. However, in the context of being solution‐oriented, the main point seemed to be that if proper equipment, education, risk evaluations and support were guaranteed, and everything was deemed to be safe for the nurses in all aspects, it would not be a problem to perform HaH:

Nurse 2 There are quite a few things that I think need to be in place first. From how the medication is transported there, to the administration, to what to do if you go to a patient who is scheduled for treatment that is not perhaps in good condition or that new problems have arisen and you need to bring in a doctor. There must be a safety net around to ensure that all aspects have been thought through.

Clearly, robust planning was seen as needed to ensure a safe HaH program. Still, many nurses were uncertain if the proper resources for performing ANPA in HaH would be available without compromising quality of the already existing care. Especially since more nurses would be needed to cover the same number of patients, many nurses believed you would only be available to treat one patient at a time in HaH, which was considered resource ineffective. In many cases, they also thought it would require robust logistics to perform HaH smoothly, especially since it would require a lot of administration to handle the scheduling of patients and replanning in case of staff shortage. Overall, the nurses believed they would need to spend more time on administration to keep the HaH service running.

Nurse 9 Without expressing any opinions on the matter, I can see certain risks in this. There are certain things in the background that could make it more difficult to see that this would work in the short term. But it also depends on the resources available. If we had ten more nurses, it would be fantastic to be able to provide home treatment. Then we could give treatment at people's homes, we could also have several people available so that it wouldn't affect this operation in the same way.

Access to healthcare software, such as the Computerized Physician order entry software (CPOE) and electronic health records (EHR), was also portrayed as a requirement to be able to access the correct information to answer patients' questions and to ‘double‐check’ prescriptions and lab values. Even though almost all nurses mentioned that it would require a lot of resources, risk analysis and planning, HaH was portrayed as something for the future, and that it could benefit the patients. Some of the nurses also viewed it as something that would be stimulating and offer new opportunities. It also seemed as if the nurses who had already worked with homecare before were more positively aligned.

Nurse 9 One of the advantages is that you can build a good relationship with patients, you can see more how patients are doing. Then you can make a better assessment of how patients are doing because you can meet the patient and see how they function in their home. I think this can be really positive! And I think it can be stimulating, to do something new and see like… well like being part of development. What I think is also good for those in my profession, because we have done very similar things for a very long time and the development is moving forward and then we nurses also have to do so, at least a little bit. So I think this could definitely be a positive aspect of it too. It could lead to becoming even better at your job too, becoming better at assessments and being able to do more assessments and do more things yourself and like see… learn more about that part, assess side effects and general well‐being of cancer patients.

## 6 Discussion

### 6.1 Reflexivity

In Box 1, some reflections on the research process are presented.

BOX 1 **Reflection on the Authors' Preunderstandings and How They Might Have Affected the Results.**

Mirroring the first author's pharmaceutical background, he expected oncology nurses to be sceptical of handling and administering ANPA in HaH settings. Hence, before the interviews started, he had practised prompts to address anticipated concerns regarding safety. He was surprised that ANPA handling in HaH environments was among the topics nurses emphasized the least and initially found it difficult to come up with suitable prompts related to the topics the nurses emphasized, such as how the patient–nurse relation would change in a HaH environment. Although the aim of the study mainly focused on nurses' handling of ANPA, the first author's pre‐understanding may have initially limited the ability to probe deeper into nursing‐specific themes, such as the nuanced ‘personal understanding’ of the patient. However, in the analysis process, the pharmaceutical background of two authors was highlighted and discussed between authors, including the second author with her non‐ pharmaceutical background and earlier experiences of both nursing research and qualitative analysis. Therefore, both pharmaceutical and nursing aspects are present in the final analysis.

### 6.2 Findings

This study aimed to investigate oncology nurses' perspectives on performing cancer treatment with ANPA in patients' homes. The main findings were that: (1) the nurses were person‐centred, and it seemed that the positive driving force was the belief that patients would experience better care in their homes. Other perspectives mentioned, such as offering a new chance to evolve within the nursing profession and a stimulating working environment, appeared to be ‘downplayed’ in relation to the patient perspective. (2) The nurses were generally concerned about working alone, mainly as the absence of colleagues in emergency situations could be stressful and challenging. (3) The nurses expressed that many parenteral ANPAs would not be suitable to use in the home because of the high risk of adverse reactions and long infusion times. (4) Concerning this, they also believed that they needed experience and education to be able to handle the policlinical care of the patient alone, without the nearby collective knowledge that the colleagues at the hospital provide. (5) Practicalities such as transporting, administering, waste handling and informing the patient about ANPA in the home seemed not to be significant barriers. In general, the nurses did not view proper handling of drugs as problematic if proper resources, support and training were given. However, some nurses believed that the home environment might be too unhygienic to prepare drugs safely and that drug waste should not be kept in the patient's home due to the risk of accidental exposure. Also, the transportation of all necessary equipment was viewed as slightly burdensome.

Overall, the nurses believed oncology HaH would be suitable if proper resources to support them were available.

#### 6.2.1 Person‐Centered Drivers and Practical Barriers in HaH

Even though the interview questions focused on the nurses' perspective on the practicalities of handling ANPA in a potential HaH program, the nurses often focused on the patient's perspective. For example, patients would get better care, as the nurses would be able to see more of the person behind the patient and dedicate more time to care for the person, patient involvement and offer emotional support. This suggests that one of the main drivers for performing HaH is anchored in a strong person‐centred care orientation. This should be taken into consideration to create an attractive and meaningful HaH programme for the nurses. Potentially by allowing additional time to facilitate a feeling of meaningfulness for the nurse and good care for the patient. Even if this would use more resources, it can lead to a reduction in cost when performing oncology patient‐centred care (Liang et al. 2020) and having dedicated time to communicate with the patient may also help the patient navigate the oncology healthcare system. The latter is important as modern treatment regimens are complex and often require patients and their families to navigate the healthcare system, potentially subjecting them to stress (Campbell et al. 2010).

The nurses pointed out that everything had to be safe and planned for them to be able to work in a home. However, they never directly expressed fear of the occupational health risk when being increasingly exposed to ANPA. This can be concerning since the nurses handle most of the drugs post‐compounding and should keep their safety in mind while doing so. Previous studies imply that oncology nurses are knowledgeable about the risks of handling ANPA, but that factors such as the safety climate of the workplace and a high workload influence the usage of safe handling routines (Callahan et al. 2016; Polovich and Clark 2012). Therefore, it might be necessary to work with the safety climate and make sure that the individual nurse has sufficient knowledge on safe handling and the consequences of exposure when moving cancer treatments to the home environment. Especially since their drug handling will not be as affected by the present collective knowledge existing at the hospital when working from home. This intertwines with the nurses' concern about working alone. We believe that this advocates the need for a framework guiding the required knowledge, experience and attributes necessary for a HaH oncology nurse to possess. What level of experience can be accepted and what tools and education should be provided to ensure a feeling of safety and not being left out while working alone as a nurse?

In addition, the nurses raised few concerns regarding the integrity of the drug, even though the drugs used in a HaH programme would undergo a different handling process than at the hospital. In oncology, many drugs are biologicals, and these are sensitive to agitation, mechanical stress and temperature changes (Martínez et al. 2023). Hence, care developers and nurses must be aware of this aspect when developing or working in HaH programs.

Many nurses expressed that providing oncology HaH would be resource inefficient compared to the hospital and feared managing adverse events alone in the home. This aligns with the result of previous research regarding oncology HaH showing that nurses reported they lacked staff, appropriate resources to manage adverse events and education to care for patients with chemotherapy (Gavin et al. 2004).

The nurses were also concerned that oncology HaH would result in more administrative work to keep the service running. A lot of the suggestions made by the nurses align with solutions used by The Christie NHS Foundation Trust outreach service (Burns et al. 2020). For example, the Trust has dedicated mobile emergency kits for spillage, adverse reactions and extravasation. Also, they report to only utilize drugs with a low risk of adverse events with short infusion times, which was also wished for by the nurses in the current study.

An earlier study has suggested that HCPs favour hospital treatment if they lack home treatment experience (Laughlin et al. 2020). In the current study, nurses without such experience were also positive to HaH, if their concerns were resolved and proper resources were available. Nevertheless, the nurses who had previously worked in homecare seemed more favourable towards performing cancer care at home. Potentially, the results from both this and the former study highlight that previous work within homecare is a valuable experience.

### 6.3 Strengths and Limitations

Several measures were taken to enhance the trustworthiness of this study, for example:

- The interview guide received external feedback and validation, to ensure the relevance of questions (Kallio et al. 2016).
- Several researchers, with different backgrounds, were involved in the data analysis, including several audits.
- The interviewer spent time in the nurses' culture to build a trustful relationship that increased the study's credibility (Amin et al. 2020).
- A reflexive research diary was used to track, explain and provide context to decisions and parts of the study (Amin et al. 2020).
- To enhance transferability, the context of the study has been thoroughly described.

The study also had limitations:

- The prescheduling of nurses could have resulted in them feeling obliged to participate and answer according to what they thought the ward chief expected, resulting in social desirability bias. However, all nurses were assured that they could withdraw from the study at any time, that their responses would remain anonymous, and that there were no correct or incorrect answers. In addition, the nurses were allowed to read through, correct and add additional clarification to the transcripts (Amin et al. 2020). That they seemed engaged during the interviews can be seen as a sign that they were personally interested in the topic and did not participate due to a felt obligation.
- Recruitment via the head‐nurse might involve a bias in who was invited. However, a majority of nurses in the ward were included in the study. As the head‐nurse was instructed to invite nurses with varied experience, and this was also seen in the final sample, it seems as though no personal bias was involved.
- The limited sample size can be considered a weakness. However, as accounted for in the method section, information power was relatively high. Although the interviewer had little experience of interviewing, possibly leading to less extensive data, the analysis showed that multiple perspectives could be seen in the material (Malterud et al. 2016).
- Data analysis was performed in Excel and by hand. There are pros and cons to using qualitative data analysis software (QDAS) versus analysing ‘by hand’. One of the main critiques of QDAS is that it can result in ‘breadth rather than depth’ and hence that the analysis becomes descriptive and/or stops at analysis rather than interpretation (St John and Johnson 2000). In this study, we chose not to use QDAS to have more in‐depth discussion, not least in the analysis steps involving several researchers. No member checking of the final themes was performed, which potentially could have added nuances to the results.
- This study was conducted in a single site, which is a limitation. The context of this site is described in the methods section to make it possible for others to evaluate the transferability to another specific context.

### 6.4 Recommendations for Future Research

This study contributes by highlighting the nurses' perspectives in oncology HaH programs, but a HaH programme is complex and requires collaboration among multiple professions. Further studies with other professions (e.g., pharmacists and oncologists) and other stakeholders would be valuable.

Since this study only included nurses who did not yet have experience with HaH, future research should investigate the perspectives of nurses who are actively performing ANPA HaH. For example, expanding on the concerns about resources and planning raised in this study, future research could quantify administrative workload, cost‐effectiveness and staffing needs. In addition, studies could investigate the practical realities of working in a home environment. This includes physical ergonomics, the experience of working alone and how nurses manage adverse events in the home.

### 6.5 Implications for Policy and Practice

The insights from this study can be used in several ways. Building knowledge and shedding light on oncology nurses' expectations of working with often hazardous, sensitive and expensive drugs in the home for treating cancer patients gives valuable insights into how to tailor oncology HaH programs to match the perceived needs of the nurses. The result can be used to create tools that tackle potential barriers and insecurities nurses might have towards managing parenteral ANPA in the patients' homes. This can potentially lead to better working conditions for the nurses and also improve patient experience and safety.

The result from the study could also, in broader terms, help decision‐makers understand the work of oncology and homecare nurses, knowledge that can be utilized when developing new services. This study can, in that sense, also contribute to raising nurses' status, showing their substantial role in cancer care.

## 7 Conclusion

Inspired by the increased interest in developing HaH oncology services, the aim of this study was to investigate oncology nurses' perspectives on performing cancer treatments with ANPA in the patient's home. Although nurses were generally positive, particularly in their belief that patients could benefit, concerns were also identified. These included the fear of managing adverse events alone; the loss of the collective knowledge and ‘safety net’ provided by colleagues, challenges related to the home environment, such as ensuring safe hygiene, achieving satisfactory work ergonomics, avoiding placing a burden on the patient and the potentially demanding organization of a HaH service. The nurses did not believe the logistics (e.g., transporting, preparing, administering and waste handling of ANPA) would be concerning, but that it would require proper planning, resources, education and support.

Working in a different context than the hospital can lead to an extended role and further responsibilities for oncology nurses and in addition, as noted by the interviewees, possibly facilitate a more person‐centred care.

## Author Contributions

**Tranberg Albin:** conceptualization, formal analysis, methodology, investigation, data curation, project administration, writing – original draft. **Kälvemark‐Sporrong Sofia:** conceptualization, formal analysis, methodology, supervision, writing – review and editing. **Paulsson Mattias:** conceptualization, supervision, formal analysis, methodology, writing – review and editing.

## Funding

The authors have nothing to report.

## Ethics Statement

Since this study did not involve any physical interventions or the collection of sensitive data, it was exempt from ethical approval from the Swedish Ethical Review Authority (Etikprövningsmyndigheten (The Swedish Ethical Review Authority) 2003: 460; Etikprövningsmyndigheten (The Swedish Ethical Review Authority), n.d.). However, ethical considerations were taken into account. Information about the study was provided to respondents, both written and oral and written informed consent was collected from the participants before the interviews started. The study includes handling personal data (demographic information about participants, audio files and transcripts); therefore, measures to follow the General Data Protection Regulation (GDPR) (Integritetsskyddsmyndigheten (The Swedish Authority for Privacy Protection's), n.d.) were applied. Hence, all interview data audio files were encrypted and stored on a USB in a locked drawer. All names or other data that could possibly identify the respondent were removed from the transcripts. Transcripts are stored on a safe server at [Uppsala University]. Prescheduling the nurses for interviews is a potential ethical weakness of this study, as they might have felt obliged to participate. Nevertheless, all participating nurses were provided with both verbal and written information that clearly outlined their opportunity to decline participation or withdraw at any time.

## Conflicts of Interest

The authors declare no conflicts of interest.

## Supporting information


**Table S1:** Example codes, underlying representative quotes and belonging demographics for the theme ‘Establishing rapport in the home environment’.
**Table S2:** Example codes, underlying representative quotes and belonging demographics for the theme ‘The complexity of a home’.
**Table S3:** Example codes, underlying representative quotes and belonging demographics for the theme ‘Adverse events – not practicalities’.
**Table S4:** Example codes, underlying representative quotes and belonging demographics for the theme ‘Solitary nursing’.
**Table S5:** Example codes, underlying representative quotes and belonging demographics for the theme ‘Planning enables performance’.

**Data S1:** Supplementary COREQ (COnsolidated criteria for REporting Qualitative research) Checklist.

## Data Availability

Research data are not shared.
