# Supplementary material for: Administering Antineoplastic Parenteral Cancer Therapy in the Patient's Home: Perspectives of Oncology Nurses—A Descriptive Qualitative Study
Source: Nurs Open. 2026 Oct 6;13(10):e70891. doi: 10.1002/nop2.70891 (PMC13639713; doi:10.1002/nop2.70891)
Supplement: Supplementary file 1 [file NOP2-13-e70891-s001.docx]

| **Theme** | **Example codes** | **Underlying quote – English translation**  (Nurse ID, quote, demographics) | **Underlying quote – Swedish original** |
| --- | --- | --- | --- |
| **Establishing rapport in the home environment** | **In the home you get to know the patient better, thereby allowing for better care** | **Nurse 6**  I really do like this job in home healthcare, that you actually get to meet the patient at home and it becomes like a different... and you see them in a different way and you gain a bit more understanding of their whole life situation and how they're doing, you can help in other ways if you see how they live or, like, how things work at home, and you can more easily discover things they might need help with and things like that, so I think you get a better picture of the patient, actually, than you do in a hospital, you don’t see everything here, but in the home you get a better overall picture, I think, absolutely! So I think you can learn a lot from that too, about the patient, and that you gain a better understanding of it  *>5 years of experience within oncology, >10 years of experience as a nurse, modest experience with homecare.* | jag tycker ändå om det här jobbet som hemsjukvård, att man faktiskt får möta patienten hemma och det blir som en annan.. och man ser dom på ett annat sätt och man får lite mer förståelse för hela deras livssituation och hur dom har det, man kan hjälpa lite på andra sätt om man ser hur dom har det eller liksom hur det funkar i hemmet och man kan lättare upptäcka saker som dom kanske behöver hjälp med och såna saker så jag tror att man får en bättre bild av patienten faktiskt än vad man får inne på ett sjukhus, man ser inte allt här, men i hemmet får man bättre sammantagen bild tror jag, absolut! så jag tror att man kan lära sig mycket av det också, om patienten och att man får en bättre förståelse för det. |
|  | **At home it is easier for the person/patient to take in information compared to the hospital** | **Nurse 3**  No, but really, maybe it could actually be a positive impact for the patient, because just the step of getting to the hospital and being in a new environment and receiving information in a new environment. I actually think that you have, you probably find it easier to take in information in your own home, but I’m speculating now, than you do with what we do here at the hospital, I think, when you come for the initial visit and there’s, like, loads of people and it’s stressful in itself just to travel here, I’d say, so I think it would probably make it easier for the patient to receive information at home, I believe so.  *<5 years of experience within oncology, <5 years of experience as a nurse, minor experience with homecare.* | Nej men egentligen kanske det skulle kunna vara en positiv påverkan för patienten för det bara ett steg att ta sig till sjukhuset och vara i en ny miljö och få information i en ny miljö så tror jag not att man har, man har nog lättare att ta till sig information i sitt hem, spekulerar jag nu, än vad man har vad vi gör här på sjukhuset tror jag, när man kommer på nybesöket och det är aaa, liksom massor folk och det är ju stressigt i sig själv att bara åka hit tänker jag, så jag tror att det nog skulle underlätta för patienten att få information i hemmet, det tror jag. |
|  | **At home it is easier for the person/patient to take in information compared to the hospital** | **Nurse 2**  How do you see yourself being affected, or that you will provide information differently in some way? Or something like that? (AT, interviewer)  Nurse 2: I almost don't think so, because we are very thorough today, but on the other hand, again, if it’s about time, then maybe to a greater extent one would have time to, like, follow up and repeat one more time, now that we’re sitting here anyway, before I leave, can we just go through this once more?, for example? I mean, it becomes a question of time, a question of time, so it’s possible that you could take it at a slower pace and explain more when you're there anyway, you might be there for two or three hours instead of running back and forth during an ongoing infusion, like you do here.  *>10 years of experience within oncology, >20 years of experience as a nurse, no previous experience of homecare.* | Hur ser du att du kommer påverkas eller att du kommer ge information annorlunda på något sätt? Eller så? (AT)  Jag tror nästan inte det för vi är väldigt noggranna idag men däremot så kanske det, igen, om det är tiden så kanske man i större grad ha tid för att liksom återkoppla och repetera en gång till, nu sitter vi ändå här och innan jag går kan vi bara gå igenom det här en gång till tex?, asså det blir en tidsfråga en tidsfråga, så att det är möjligt att man skulle kunna ta det i lugnare takt och förklara mera när man ända är där man är i kanske två tre timmar istället för att man springer hit och dit under pågående infusion som man gör här. |

**Supplementary Table S1:** Example codes, underlying representative quotes and belonging demographics for the theme “Establishing rapport in the home environment”.

| **Theme** | **Example codes** | **Underlying quote – English translation**  (Nurse ID, quote, demographics) | **Underlying quote – Swedish original** |
| --- | --- | --- | --- |
| **The complexity of a home** | **It is important that the home is sufficiently hygienic to work in.** | **Nurse 4**  In terms of the actual work environment, it might not be that easy, it also depends on how the patient... not all homes look the same... it can be cramped, and it can be dirty, and there can be pets, you know... everything, pets, a cat that thinks that tube there looks a bit fun...  *>10 years within oncology, >20 years of experience as a nurse, no previous experience of homecare.* | rent arbetsmiljömässigt så kanske det inte är så lätt, det beror ju på också hur patienten... alla hem ser ju inte lika ut.. det kan ju vara trångt och det kan vara smutsigt och det kan vara husdjur, du vet... allting, husdjur, en katt som tycker den där slangen ser lite rolig ut... |
|  | **Work ergonomics can be complicated in the home setting compared to the hospital** | **Nurse 7**  Would you like to elaborate on that part about the work environment? (AT, interviewer)  Nurse 7: Yeah, I’m thinking more about how you stand and sit when you’re inserting needles and, ummm, sometimes you might need something... you might give these subcutaneous injections of 11 ml, and then you feel like your back starts to hurt after 10 minutes if you have to sit there in a pretty static angle, sort of.  *>10 years within oncology, >20 years of experience as a nurse, no previous experience of homecare.* | Vill du utveckla just den biten med arbetsmiljö? (AT)  SSK: Jag jag tänker väll mer på hur man står och sitter när man ska sätta nålar och ehmmm, ibland så kan man behöva någonting... man kan ju ge såna här subkutana injektioner på 11 ml och då känner man ju att man får lätt ont i ryggen i 10 minuter om man ska sitta där i en ganska statisk vinkel liksom |
|  | **Cancer care at home requires a lot of equipment that must be flexible and portable** | **Nurse 2**  But I think it would work well. All the equipment must, of course, be in place so you can give the IV drip at the right rate, and even when there are a lot of other things surrounding each patient to insert a peripheral venous catheter needle, you need a dressing kit, you need alcohol, you need gloves, and you need syringes, you need saline. It’s also that thing about being able to practically carry it all around with you.  *>10 years of experience within oncology, >20 years of experience as a nurse, no previous experience of homecare.* | men jag tror det skulle funka bra, all utrustning måste förstås finnas på plats så man kan ge dropp i rätt takt och även när det är mycket andra prylar omkring varje patient, för att sätta en venportsnål måste du ha ett omläggningsset, du behöver sprit, du behöver ha handskar och du behöver ha sprutor du behöver ha koksalt. det är också det där med att man praktiskt kan ha med sig det runt |

**Supplementary Table S2:** Example codes, underlying representative quotes and belonging demographics for the theme “The complexity of a home”.

| **Theme** | **Example codes** | **Underlying quote – English translation**  (Nurse ID, quote, demographics) | **Underlying quote – Swedish original** |
| --- | --- | --- | --- |
| **Adverse events – not practicalities** | **Cancer treatments involve unpredictable adverse reactions that one does not want to face in the home** | **Nurse 1**  From our perspective, I see concerns. Because now, I don’t think there were any alarms while you were here, but usually there are, quite often and being at home and administering very potent drugs, even if it’s an antibody, it’s associated with a risk of the patient reacting, that’s the case with all medications, of course. But even more so with these.  *>10 years of experience within oncology, >20 years of experience as a nurse, no previous experience of homecare.* | Ur vårat perspektiv så ser jag farhågor, därför att, nu vet jag inte särskillt inget larm när du var här, men det är ju det vanligtvis ganska ofta och att vara hemma och administrera väldigt potenta läkemedel även om det är en antikropp, så är det ju förent med risk för att patienten ska reagera, det är ju så med alla läkemedel förstås. Men ännu mera med de här. |
|  | **It is important to provide medications that do not lead to reactions, and to have sufficient experience.** | **Nurse 8**  Then it is very much dependent on which treatment you give, if we disregard what I just said and imagine that we are going to do it anyway, how that would feel. But in any case, I would personally need even more experience, and we should not give medications that often cause reactions, because for those you need to have emergency preparedness  *<5 years of experience within oncology, >10 years of experience as a nurse, no previous experience of homecare.* | Sen är det ju väldigt beroende på vilken behandling man ger om man bortser från det jag precis sa och tänker att vi ändå ska göra det, ehmm hur det skulle kännas... Men jag skulle i alla fall behöver ännu mer erfarenhet och att man inte ger läkemedel som man ofta reagerar på ehmm, för dom behöver man ha akutberedskap på |
|  | **Cancer treatments involve severe and unpredictable adverse reactions that can make them unsuitable for the home.** | **Nurse 3**  Mmmm, and when you say 'the treatment can bring things with it,' what do you mean by that? (AT, interviewer)  Nurse 3: Well, what I mean is that you can get allergic reactions, you can get side effects, in the worst-case scenario, you can have a cardiac arrest. Uhm, you never really know how patients will react, you have, roughly, an idea of how they will react based on how it has been before. Uhm, but reactions can occur that you don't think will happen, and the patient can feel much worse when you think they should be better, which is why it is unpredictable. Uhm, those are the problems as I see them.  *<5 years of experience within oncology, <5 years of experience as a nurse, minor experience with homecare.* | mmmm, och när du säger ”behandlingen kan föra med sig”, vad menar du då? (AT) Då menar ju jag att man kan få en allergisk reaktioner, man kan få biverkningar, i värsta fall kan man få hjärtstopp . ehhmmm du kan ju, du du vet ju aldrig egentligen hur patienterna reagerar, man har ju, på ett ungefär, så har man ju som man vet hur de kommer reagera ut efter hur det har varit tidigare. Ehhmmm men det kan ju uppstå reaktioner som man inte tror ska uppstå och patienten kan må mycket sämre som man tror ska vara bättre, därför är det ju oförutsägbart. ehmmm det är ju det som är dom här problemen som jag ser det då. |

**Supplementary Table S3:** Example codes, underlying representative quotes and belonging demographics for the theme “Adverse events – not practicalities”.

| **Theme** | **Example codes** | **Underlying quote – English translation**  (Nurse ID, quote, demographics) | **Underlying quote – Swedish original** |
| --- | --- | --- | --- |
| **Solitary nursing** | **The vulnerability of working alone in a home when something goes wrong and emergency assistance is lacking** | **Nurse 1**  And that’s when it becomes a problem, because who are we going to blame if we’ve given the patient something they react to and then you don’t manage to reverse it, and... the ambulance arrives after 10 minutes, by then, it’s too late. That’s how fast it goes, because sometimes it only takes a few minutes, and I don't want to be that nurse standing there, honestly. Here, you have an alarm button you can press and then every nurse working here comes at once, there’s no doubt about it, then we are 10 people standing around the patient, and the on-call doctor is alerted and arrives in the blink of an eye, but we’re still 10 people who can help each other and perform CPR, because that has happened several times.  *>10 years of experience within oncology, >20 years of experience as a nurse, no previous experience of homecare.* | .  och då blir det problem, för vem ska vi skylla på om vi har gett patienten något att reagera på och sen så lyckas man inte häva ååå och ambulansen kommer efter 10 minuter, då är det ju kört. Så fort går det för det tar några minuter ibland, och jag vill inte vara den sköterskan som står då, faktiskt. Här har du en larmknapp du kan ringa på, du får alla sjukskköterksor som jobbar här kommer på en gång, det är ingen tvekan om det, då är vi 10 pers som står runt patienten och jouren larmas och kommer snabbt som ögat men vi är ändå 10 personer som kan hjälpas åt och ge hjärtmassage, för det har hänt flera gånger. |
|  | **The need for a colleague nearby for consultation and support during potent treatments at home** | **Nurse 6**  But at the same time, it’s always nice to have someone who you have here. We also work somewhat independently, but you always have someone to consult and you always have someone to... which you have, we also work somewhat independently, but you always have someone to consult, you always have someone to bounce ideas off of, “what do you think?”, And if you’re going to make an assessment, it’s good if someone else can come and take a look so that... purely in terms of giving these potent drugs at home, it would be nice to at least be two people who are at least driving together, or that you’re maybe close by, at least, so you can reach a colleague within a short time, reach a colleague quickly who can come and help out if needed. I think that it would be necessary for that type of thing, if we were to give these treatments!  *>5 years of experience within oncology, >10 years of experience as a nurse, modest experience with homecare.* | men samtidigt är det alltid skönt att ha någon vilket man har här, vi jobbar ju också lite självständigt men man har ju alltid någon att rådfråga och man har alltid någon att vilket man har, vi jobbar ju också lite självständigt men man har ju alltid någon att rådfråga, man har alltid någon att bolla med, "vad tror du?" och om man ska göra en bedömning så är det bra om någon annan kan komma och titta så att... rent om man ger så här potenta läkemedel i hemmet så skulle det ju vara skönt att åtminstonde vara två som åtminstonde kör ihop eller att man kanske på nära håll, åtminstode, så man kan nå någon kollega inom kort tid så man kan nå någon kollega på kort tid som kan komma och hjälpa till om det skulle behövas. det tror jag skulle behövas, det är den typen om man skulle behöva ge dom här behandlingar.! |
|  | **Handling acute situations alone at home is an added responsibility** | **Nurse 2**  What’s added, I think, is more about if something more acute happens. If something acute happens here, then a couple of nurse colleagues stream in and the on-call doctor can be there in 5 minutes. That’s not how it will be if you’re at home with someone who maybe lives... especially if the patient who lives a bit further away has the greatest need for this service. So I’m thinking that it becomes like a greater responsibility in that situation for the nurse alone to be able to handle it while waiting for someone else to show up, mm mm, but otherwise I don't think there will be that much of a difference in that sense.  *>10 years of experience within oncology, >20 years of experience as a nurse, no previous experience of homecare.* | det som tillkommer  tycker jag handlar ju mer om det händer något mer akut, att ehhhmmm det om det händer något akut här då strömmar det till ett par sjuksköterskor kollegor och jouren läkaren kan vara där på 5 minuter, så blir det inte om fallet är att man är hemma hos någon som kanske bor kanske också framförallt om patienten som bor lite bort har störst behov av den här tjänsten , ehmmm så jag tänker att det blir liksom ett större ansvar i en sån situationen på sjuksköterskan själv att kunna hantera det i väntan på någon annan kan dyka upp mm mm, men i övrigt tror jag det inte blir så stor skillnad rent så. |

**Supplementary Table S4:** Example codes, underlying representative quotes and belonging demographics for the theme “Solitary nursing”.

| **Theme** | **Example codes** | **Underlying quote – English translation**  (Nurse ID, quote, demographics) | **Underlying quote – Swedish original** |
| --- | --- | --- | --- |
| **Planning enables performance** | **The practical logistics of medication transport**  **are viewed as a manageable task.** | **Nurse 3**  Nurse 3: Yeah, but that would work well, absolutely! I don't see any problem with that, having to transport the treatment itself, that would work fine."  Yeah, I understand. Would you like to elaborate on that, specifically? (AT, interviewer)  Respondent: Nooo, or I’m not really sure what you’re referring to... are you thinking that there... I mean, that there would be some problem with the administrations when you’re driving the medications? For me, I’m just thinking, ahh, well, you probably have a box with you, and you bring the medications along, if it needs to be refrigerated, you’d have a cooler or something like that. So, I don’t really see any problems with transporting the medication as such, because as long as it’s prepared like it is today and you just pick it up, it feels like a fairly simple task anyway.  *<5 years of experience within oncology, <5 years of experience as a nurse, minor experience with homecare.* | .  aaaa, aaa (AT)  Ja men det skulle funka bra, absolut! Det ser jag inget problem med, att man ska frakta själva behandlingen, det skulle funka bra.  aa jag förstår, Vill du utveckla, just liksom? (AT)  neeej , eller jag vet inte riktigt vad du syftar till, tänker du att det , asså det skulle var något problem med administeringar när man kör läkemedlerna? För mig tänker jag bara ahh men att man har väll en box med sig och så tar man med sig läkemedel, behöver det vara kylt får man ha en kylbox eller sådär, och så jag ser egentligen inga problem med att frakta själva läkemedlet som sådant, för så länge det är berett som det är idag och så hämtar man bara det så känns det som en ganska simple task ändå. |
|  | **Education and safety standards are needed for home-based drug preparation** | **Nurse 5**  It wouldn’t be any problem for me... I wouldn’t have any problem doing it [preparing/mixing drugs] if that was the alternative, as long as I knew how to do it... provided one receives training in how to do it if it’s something the pharmacy usually mixes and we are to learn to mix it ourselves, for example. As long as it isn't a danger to me to do it.  *<5 years of experience within oncology, <10 years’ experience as a nurse, minor experience of homecare.* | Det skulle jag inte ha heller några problem med bara jag visste hur jag skulle... om man får en... man får utbildning i hur man ska göra om det är något liksom som beredningsenheten vanligtvis blandar och vi ska lära oss att blanda till exempel. Och så länge det inte är någon fara för mig att göra det liksom... hade inte jag haft några problem att göra det om det var det som var alternativet liksom. |
|  | **Improved nursing competency and patient insights in the home setting.** | **Nurse 9**  One of the advantages is that you can build a good relationship with patients, you can see more of how patients are doing, then you can make a better assessment of how patients are doing because you can meet the patient and see how they function in their home. I think this can be really positive! And I think it can be stimulating to do something new and see like... well but like being part of development. What I think is also good for those in my profession, because we have done very similar things for a very long time and the development is moving forward and then we nurses also have to do so, at least a little bit. So I think this could definitely be a positive aspect of it too. It could lead to becoming even better at your job too, becoming better at assessments and being able to do more assessments and do more things yourself and like see... learn more about that part, assess side effects and general well-being of cancer patients.  *<5 years of experience within oncology, <10 years’ experience as a nurse, minor experience of homecare.* | Men... som för oss sjuksköterskor... det är ju det som är en fördel, att man kan få en bra relation med en patient... så att man verkligen ser ännu mer hur patienten mår då. Man kan göra en bättre bedömning när man får träffa dem i deras hem och se hur de fungerar... i sitt hem, det tror jag kan vara jättepositivt. Det är rent stimulerande att få liksom... göra någonting nytt och vara med i utvecklingen, det tror jag är väldigt bra för min yrkesroll också. Man har gjort väldigt lika saker under väldigt lång tid... och utvecklingen går framåt, det måste vi också göra lite grann. Det tror jag absolut skulle kunna vara en positiv aspekt också... att man blir ännu bättre på sitt jobb, att man blir duktigare på bedömningar när man får göra mer själv och liksom lära sig ännu mer om den biten... att bedöma biverkningar och hur de mår generellt hos den här patientgruppen, alltså cancerpatienter… |

**Supplementary Table S5:** Example codes, underlying representative quotes and belonging demographics for the theme “Planning enables performance”.
