# Supplementary material for: Administering Antineoplastic Parenteral Cancer Therapy in the Patient's Home: Perspectives of Oncology Nurses—A Descriptive Qualitative Study
Source: Nurs Open. 2026 Oct 6;13(10):e70891. doi: 10.1002/nop2.70891 (PMC13639713; doi:10.1002/nop2.70891)
Supplement: Supplementary file 2 [file NOP2-13-e70891-s002.docx]

# Supplementary COREQ (COnsolidated criteria for REporting Qualitative research) Checklist

A checklist of items that should be included in reports of qualitative research. You must report the page number in your manuscript where you consider each of the items listed in this checklist. If you have not included this information, either revise your manuscript accordingly before submitting or note N/A

S = Section in the manuscript, for example S4.3.2 = “4.3.2 Data collection”

| **Item No** | | **Guide Questions/Description** | **Reported on Page #** |  |  |
| --- | --- | --- | --- | --- | --- |
| **Domain 1: Research team and reflexivity** | | | |  |  |
| **Personal Characteristics** | | | |  |  |
| 1. Interviewer/ facilitator | | Which author/s conducted the interview or focus group? | 7 (S4.3.2) |  |  |
| 2. Credentials | | What were the researcher’s credentials? E.g., PhD, MD | 5 (S4.1) |  |  |
| 3. Occupation | | What was their occupation at the time of the study? | 5 (S4.1) |  |  |
| 4. Gender | | Was the researcher male or female? | 5 (S4.1) |  |  |
| 5. Experience and training | | What experience or training did the researcher have? | 5 (S4.1) |  |  |
| **Relationship with participants** | | | |  |  |
| 6. Relationship established | | Was a relationship established prior to study commencement? | 6 (S4.2) |  |  |
| 7. Participant knowledge of the interviewer | | What did the participants know about the researcher? e.g. personal goals, reasons for doing the research? | 6 (S4.2) |  |  |
| 8. Interviewer characteristics | | What characteristics were reported about the interviewer/facilitator? e.g. Bias, assumptions, reasons and interests in the research topic | 5(S4.1), 15 (S6.1) |  |  |
|  | **Domain 2: study design** | | |  |  |
|  | **Theoretical framework** | | |  |  |
|  | 9. Methodological orientation and Theory | What methodological orientation was stated to underpin the study? e.g. grounded theory, discourse analysis, ethnography, phenomenology, content analysis | 8 (S4.4) |  |  |
|  | **Participant selection** | | |  |  |
|  | 10. Sampling | How were participants selected? e.g., purposive, convenience, consecutive, snowball | 5 - 6 (S4.2) |  |  |
|  | 11. Method of approach | How were participants approached? e.g., face-to-face, telephone, mail, email | 6 (S4.2) |  |  |
|  | 12. Sample size | How many participants were in the study? | 9 (S5.1) |  |  |
|  | 13. Non-participation Setting | How many people refused to participate or dropped out? Reasons? | 9 (S5.1) |  |  |
|  | 14. Setting of data collection | Where was the data collected? e.g., home, clinic, workplace | 7 (S4.3.2) |  |  |
|  | 15. Presence of nonparticipants | Was anyone else present besides the participants and researchers? | N/A, clarified at  7 (S4.3.2) |  |  |
|  | 16. Description of sample | What are the important characteristics of the sample? e.g. demographic data, date | 9 (S5.1) |  |  |
|  | **Data collection** | | |  | No |
|  | 17. Interview guide | Were questions, prompts, and guides provided by the authors? Was it pilot tested? | 6-7 (S4.3.1) |  |  |
|  | 18. Repeat interviews | Were repeat interviews carried out? If yes, how many? | N/A, clarified at 7 (S4.3.2) |  |  |
|  | 19. Audio/visual recording | Did the research use audio or visual recording to collect the data? | 7 (S4.3.2) |  |  |
|  | 20. Field notes | Were field notes made during and/or after the interview or focus group? | 7 (S4.3.2) |  |  |
|  | 21. Duration | What was the duration of the interviews or focus group? | 7 (S4.3.2) |  |  |
|  | 22. Data saturation | Was data saturation discussed? | 8-9 (S4.5) |  |  |
|  | 23. Transcripts returned | Were transcripts returned to participants for comment and/or correction? | 7 (S4.3.2) |  |  |
|  | **Domain 3: analysis and findings** | | |  |  |
|  | **Data analysis** | | |  |  |
|  | 24. Number of data coders | How many data coders coded the data? | 8 (S4.4) |  |  |
|  | 25. Description of the coding tree | Did the authors provide a description of the coding tree? | 8 (S4.4), Supplementary material-table1 |  |  |
|  | 26. Derivation of themes | Were themes identified in advance or derived from the data? | 8 (S4.4) |  |  |
|  | 27. Software | What software, if applicable, was used to manage the data? | 8 (S4.4) |  |  |
|  | 28. Participant checking | Did participants provide feedback on the findings? | N/A, clarified at 8 (S4.4) |  |  |
|  | **Reporting** | | |  |  |
|  | 29. Quotations presented | Were participant quotations presented to illustrate the themes/findings? Was each quotation identified? e.g., participant number | See the findings section, 10,11,12,13,14,15 |  |  |
|  | 30. Data and findings consistent | Was there consistency between the data presented and the findings? | See the findings section, 10,11,12,13,14,15 |  |  |
|  | 31. Clarity of major themes | Were major themes clearly presented in the findings? | See the findings section, 10,11,12,13,14,15 |  |  |
|  | 32. Clarity of minor themes | Is there a description of diverse cases or a discussion of minor themes? | 8 (S4.4), 10-15, |  |  |
